# Memoir on the Cause of Hunger and Thirst

**Published:** 1803-07-01

**Authors:** C. L. Dumas

**Affiliations:** M. N. I. Professor of Anatomy and Physiology, in the University of Montpelier


MEMOIR *on the* CAUSE *of* HUNGER *and* THIRST, *by* C. L. DUMAS, *M. N. I. Professor of Anatomy and Physiology, in the University of Montpelier, and lately communicated to the Medical Society of that Town.*

PHYSIOLOGISTS, says the author, have long looked for the proximate cause of hunger, and they have alternately attributed it to mechanical, physical, and chemical causes; to overturn all of which, it is only necessary to oppose the influence of habit, imagination, and will.

The friction of the membranes of the stomach in a state of emptyness; the compression of the nerves occasioned by the sinking in of the muscular fibres; the increased volume of the liver and spleen, owing to the afflux of blood in the splenic and hepatic vessels; the increase in quantity and acrimony of the digestive liquors, such were the explanations given to this sensation. The author observes, that however empty the stomach, its parietes cannot move or produce any friction so as to become painful; and he asks, what resemblance can this motion produce to the constant sensation arising from a want of nourishment? And though this sensation be produced by friction, he asks, how it happens that the quantity and choice of our aliment becomes limited? It should not, he conceives, be directed to the use of such or such a substance, to the exclusion of every other; and hence he concludes, that a physical or mechanical cause can never explain that taste or preference connected with this sensation.*

The

* We are as little disposed to admit a mechanical or physical explanation of hunger as the author; but we cannot think him happy in the objections he brings against it, in as far as that taste or preference for particular food, as well as the limits of our appetites, depend for the most part on education, experience, and habit.

The same refutations, he thinks, overturn the opinions that hunger arises from a chemical cause, as the acrimony of the humours, &c. Admitting such explanations, we should ask, why we feel the sensation of hunger at particular hours? Why all those causes which give a new direction to our sensibility should suspend it, &c.?

The author thinks that the explanations given of thirst are not more satisfactory. This sensation is increased by all those causes which deprive the blood of its aqueous humours. The accumulation of these fluids in the great cavities, when the lymphatics are unable to re-absorb and throw them again into the mass of blood, is another powerful cause which increases the intensity of this sensation. Dropsical people reckon this among the most tormenting of their symptoms. Inflammatory diseases, but particularly inflammations of the viscera, are attended by thirst. The same circumstance characterises febrile diseases, hæmorrhagies, and all those that seem particularly to affect the vascular system.

The effects of thirst act particularly on this system, which is proved not only by the redness of the lips and tongue, the dryness of the palate, fauces, and throat, but also by the thickening and inflammatory disposition of the blood. Though the sensation of thirst increases rapidly, still it is supportable for a much longer time than that of hunger. The examples of abstinence from drink for a number of days or months are sufficiently numerous. After reflecting on the phenomena of hunger and thirst, I soon perceived, continues the author, the insufficiency of the hypothesis which I have just mentioned; I occupied myself daily in searching for an explanation more satisfactory. I tried to form an opinion free from all conjecture, and which should tally with those facts which I had been at considerable pains to collect; and, lastly, I wished to support my notions by some experiments which should establish their truth.

Hunger and thirst, says the author, suppose certain modifications of the nervous system; but here, as well as in other kinds of painful sensations, they are not always owing to the same cause, nor are they produced by some original alteration; hunger he conceives to relate more to the lymphatic system, thirst to the vascular; one is connected with a penury of nutritious fluids, the other with a superabundance; the first shews a diminution of force, the second an increase; the former is asthenic, the latter sthenic. In hunger, the absorbents act on the sensitive system. The blood-vessels act on this system in thirst. Aliments become necessary to the renewal of the different substances of the body, as well as its forces, and to increase in the fluids the quantity of albuminous and fibrous parts. Drink becomes necessary to moisten the solids, to dilute the fluids, to temper the vital energy, to remedy the too great thickening, &c. of the concrete parts of the blood. The phenomena of hunger, and the great influence this has on the habits and passions, prove that it is a nervous affection immediately dependant on sensibility. Wounds of the brain and ligatures of nerves bring on a loss of appetite. Whatever engages the mind, whether painful or otherwise, suspends the sensation of hunger. The author tells us that these several considerations first suggested to him the idea, that hunger was but a modification of the nervous system; but the nervous system is closely connected with the lymphatic; and he conceives the same proofs are in support of his second notion, that hunger particularly affects the lymphatic system. These considerations he thought sufficiently interesting to merit further inquiry; and the author gives an account of some experiments which tend to throw light on his doctrine.

Exp. 1. A large dog had been deprived of all kind of aliment for eight days, during which time small quantities of opium were administered. At each dose, and for some hours after, the animal ceased to complain.

Exp. 2. A similar dog was made to fast for several days; he became furious; a mixture of opium and camphor was thrown to him, which he devoured immediately, after which he became more calm, which the author availed himself of to give the same mixture in smaller doses; by degrees the animal's hunger was so completely appeased, that he refused nourishment when offered.

Exp. 3. The author gave, in large quantity, oil emollient decoctions, warm water, &c. to a number of dogs, who had not received food for a length of time; these means, though they diminished for a moment the torments of the animals, yet they soon returned with an increase. If instead of these relaxing substances, tonics, spirits, aromatics, or cold water be given, the effect is different; the hunger is appeased, and the power of abstinence prolonged.

Exp. 4. A solution of super-oxygenated muriat of mercury, given in moderate quantity, was found to diminish hunger; and dogs, to whom this substance had been often administered, were observed to lose their appetite. The author thor concludes from these experiments, that opium, spirits, aromatics, mercurials, &c. not only prevent hunger, but also appease it. The activity of the absorbent system is likewise diminished by the same substances; the same causes, he observes, act in the same way, and produce the same effects on the lymphatic system and on hunger, there must therefore be an intimate connection between the two; and which seems to be further proved by the following experiment.

Exp. 5. Four dogs, of the same size and age, were taken, and deprived for many days of solid food. Three were killed at separate periods, the fourth was permitted to die of hunger. On opening the first, the stomach was found contracted, the viscera displaced, and a quantity of drink, which had been given a short time before his death, was entirely absorbed. The appearances in the second were similar, except that the proper liquors of the stomach, the gastric juice, &c. were not found in the usual quantity, being in a great measure absorbed. In the third, all the gastric and pancreatic juices, all the mucus of the intestines, and even the serous fluid of the peritoneum were absorbed, all the viscera of the abdomen was shrunk up. In the fourth, the appearances seemed to indicate that the absorbents had begun to act on the solids themselves; the inner surface of the intestines seemed to be attacked in many points, the absorbent vessels were seen exposed, and their power of absorption continued a long time after the death of the animal.

These facts, the author adds, unite with the observations of practitioners in shewing the concurring influence of the nervous and lymphatic systems on the sensation of hunger; they also prove the asthenic nature of this affection, and serve to elucidate the causes of hunger. M. Dumas observes, 1st. That opium diminishes appetite but increases thirst. 2d. That spirits rather irritate than appease thirst. 3d. Fever from artificial excitement, as well as fever from local inflammation, were always followed by thirst. 4th. Having appeased the hunger of a dog without permiting him to drink, and after suffering from thirst a long time, which at length became excessive, a saturated solution of nitrate of potash in water was given to him; his thirst became immediately quieted. The same experiment was repeated on two other dogs; to one, a quantity of pure water was given, of which he drank a great quantity, and a considerable time was necessary before his desire ceased; a pint of the solution of nitre in water was sufficient ent to content the other. Nitrous salts, the author observes, debilitate the vascular system, and diminish its action, and for which reason they are useful in fevers, where the febrile state in general is confined to this system. I found, says M. Dumas, that bleeding often, and in small quantity, alleviated them; and he thinks, as well from observation as from experience, that thirst as well as hunger, is an affection of the nervous system, but owing to a very different cause; and that these two sensations have, with respect to the animal body, a very different object and end. I have endeavoured however, he says, to determine by one other experiment more precisely my ideas on the subject; and in consequenee of which, I do not hesitate to conclude that thirst is a nervous sensation arising from the influence of the vascular system, and partaking of the sthenic or inflammatory character.

A number of dogs were deprived of drink; such food only was given as was capable of irritating them; when their sufferings from thirst were at the highest pitch, a large quantity of blood was taken from one of them; thirst was for the moment quieted, but what appeared more important, was, that the blood threw up a thick inflammatory crust. Another dog was killed in the midst of his torments, and the marks of inflammation were evident on most of the viscera; some parts seemed to be threatened by gangrene, and the heart and vessels were full of a thick coagulated blood, as is observed in the neighbourhood of inflamed parts. The author concludes by telling us, that he had undertaken a set of experiments with a view to determine what were the best means of exciting hunger and thirst; but he hesitates to assert that it will be found that hunger is excited by every direct stimulus of the nervous and lymphatic systems, while thirst will be produced by stimulating the vascular. A variety of considerations and objections to our author's conclusions and reasoning will arise in the mind of the intelligent physiologist on perusing this Memoir, and which it is unnecessary to suggest. The experiments, however, are not without interest.

We only think it useful to observe, that M. Dumas is author or rather compiler of a very elaborate System of Physiology, and that, as a professor and practitioner, he enjoys the first character in the Medical School of Montpelier.

